# Do patient surveys work? The influence of a national survey programme on local quality-improvement initiatives

**DOI:** 10.1136/qshc.2007.022749

**Published:** 2008-11-26

**Authors:** R Reeves, I Seccombe

**Affiliations:** Healthcare Commission, London, UK

## Abstract

**Objectives:**

To assess current attitudes towards the national patient survey programme in England, establish the extent to which survey results are used and identify barriers and incentives for using them.

**Design:**

Qualitative interviews with hospital staff responsible for implementing the patient surveys (survey leads).

**Setting:**

National Health Service (NHS) hospital organisations (trusts) in England.

**Participants:**

Twenty-four patient survey leads for NHS trusts.

**Results:**

Perceptions of the patient surveys were mainly positive and were reported to be improving. Interviewees welcomed the surveys’ regular repetition and thought the questionnaires, survey methods and reporting of results, particularly inter-organisational benchmark charts, were of a good standard. The survey results were widely used in action planning and were thought to support organisational patient-centredness. There was variation in the extent to which trusts disseminated survey findings to patients, the public, staff and their board members. The most common barrier to using results was difficulty engaging clinicians because survey findings were not sufficiently specific to specialties, departments or wards. Limited statistical expertise and concerns that the surveys only covered a short time frame also contributed to some scepticism. Other perceived barriers included a lack of knowledge of effective interventions, and limited time and resources. Actual and potential incentives for using survey findings included giving the results higher weightings in the performance management system, financial targets, Payment by Results (PbR), Patient Choice, a patient-centred culture, leadership by senior members of the organisation, and boosting staff morale by disseminating positive survey findings.

**Conclusion:**

The national patient surveys are viewed positively, their repetition being an important factor in their success. The results could be used more effectively if they were more specific to smaller units.

The stated aims of patient feedback programmes are normally twofold: to monitor performance and to stimulate improvements in the quality of care. These goals are not contradictory, but neither are they entirely complementary. Patient experience surveys are now widely accepted as valid indicators of healthcare performance, but their usefulness in improving the quality of care at the organisational level has not yet been systematically researched.

In England, the Healthcare Commission is now responsible for the National Health Service (NHS) patient experience survey programme, as set out in the *NHS Plan*.^1^ Since 2002, a programme of annual surveys in NHS hospitals in England has covered inpatients, emergency departments, outpatients and young patients aged 0–18. For each eligible NHS trust, 850 recently treated patients are sampled. For inpatient and young patient surveys, consecutively discharged patients are sampled, counting back from an agreed date, so, for larger trusts, the sampling period can be less than 1 month, while surveys in trusts with lower volumes of patients cover periods of up to 6 months. For outpatients and emergency surveys, random samples of patients are taken from 1 month’s de-duplicated attendances. Questionnaires comprising 50–100 closed questions and a space for written comments are posted to selected patients, and up to two reminders are sent to non-responders at 2-weekly intervals. Using detailed written guidance, each survey is conducted according to a standard protocol by an approved contractor on the trust’s behalf or, in a small and falling proportion, by the trust itself.

Benchmarks of trusts’ performance on each of the survey questions have been published and the results used to calculate the Healthcare Commission’s “patient focus” performance indicators. Most surveys have been repeated, so longitudinal data are available. National results published in key findings reports^2^^–^5 show that, at the national level, most aspects of experience have not changed over time, but there are a few improvements where central government has set targets.

## ATTITUDES TOWARDS PATIENT SURVEYS

In a number of countries, healthcare workers’ attitudes towards patient experience surveys and patient feedback reports in general have been found to be broadly positive.^6^^–^9 In a UK study, the most useful sources of patient experience information were thought to be complaints, comments and compliments, patient surveys, and information from the Patient Advice and Liaison Services (PALS).^9^ In one study, patients’ written comments were valued more highly than survey scores.^6^

There is also evidence that some healthcare workers are sceptical about the value of surveys. Criticisms include concerns that questionnaires are too lengthy, and that the surveys are not cost-effective.^10^

## EFFECTS OF PATIENT SURVEYS

The evidence that patient surveys can stimulate local quality improvements is equivocal. UK healthcare leaders claimed their NHS trust had made positive changes following from patient surveys,^9^ and a US study in 90 hospitals found several improvements following the issue of patient reports.^11^ In a French hospital, clinicians thought patient surveys made the culture more patient-centred but did not lead to systematic improvement.^6^ However, a number of studies have detected little or no improvement in care quality following from patient survey programmes.^10^ ^12^ ^13^ A systematic review concluded that audit and feedback data usually have small to moderate effects on health professionals’ practice.^14^

## BARRIERS AND DRIVERS

The perceived barriers to using patient survey results include a lack sufficiently specific information at the level of smaller units within healthcare facilities;^9^ ^10^ ^15^ delays in disseminating results;^9^ ^15^ lack of expertise or knowledge of effective interventions;^9^ ^15^ limited understanding of statistical methods;^15^ lack of time for clinical teams to discuss the results; and the low priority given to using survey results within the NHS and scepticism among clinicians about the validity of the surveys.^9^

Suggested ways of improving the use of survey findings include “a systematic approach to quality improvement;” giving survey results higher weightings in the performance management system;^9^ leadership by senior members of the organisation,^9^ ^15^ organisational support for leaders in quality improvement and training staff in quality improvement methods.^16^ Public reporting of results and the preservation of detailed information about survey results (as opposed to aggregated “report cards”)^17^ ^18^ and reporting results at the level of smaller units^16^ have also been cited as important factors in the success of patient survey programmes.

## FOCUS OF THIS STUDY

The aim of this research is to document current attitudes towards the national patient survey programme, to establish the extent to which patient survey results are currently used in NHS hospitals and to identify the drivers and barriers experienced by NHS staff in using survey results.

## METHODS

### Selection of organisations

Twenty-seven hospital organisations (NHS trusts) were selected from the 169 NHS trusts providing adult acute services that were in existence in England in spring 2006. Trusts were selected purposively, with the aim of covering a broad range of performance scores, size and geographical spread within England. The proportions of selected trusts was approximately representative of four subgroups (divided by trust size and location in London or outside London) within the total population: two of the 13 large London trusts were selected, nine of the 55 large outside London trusts, three of the 19 medium/small London trusts and 13 of the 82 medium/small outside London trusts. The selected trusts were also broadly representative of the range of performance ratings across the population. Of the 69 trusts with the highest three-star rating in the Healthcare Commission’s 2005 performance ratings, 14 were selected for inclusion; seven of the 53 two-star trusts, five of the 38 one-star trusts and one of the nine zero-star trusts were also selected.

### Interview procedure

In the first quarter of 2006, in the 27 selected trusts, telephone calls were made to the person listed in Healthcare Commission records as the lead for patient surveys. Before the interview started, the interviewer checked that the contacted person had primary responsibility for responding to patient survey results and if someone else had this responsibility instead, the researcher contacted that alternative person. Semistructured interviews were conducted by one interviewer using an interview guide which was based on the reviewed literature and discussions with opinion leaders in the uses patient survey results. Interviewees were asked about their uses of the patient surveys; their views on the quality of the questionnaires, survey methods and reporting of results; and factors that facilitated or hindered their using survey results. Participants had an opportunity to review and correct the interview notes.

### Analysis procedure

The verified interview notes were manually coded and categorised, and initial themes were identified by the interviewer. After discussion between both researchers, the themes were modified and reduced by merging them.

## RESULTS

### Characteristics of participating organisations and interviewees

#### Organisations

Interviews were successfully completed at 24 of the 27 trusts. The three non-participating trusts were all medium/small outside London; two of them had two stars, and one had one star. Efforts to arrange interviews with people from the non-responding trusts were stopped after at least five attempts had been made to identify an appropriate staff member, but none of those trusts actively refused to participate in the research, or gave a reason for not wanting to participate.

#### Interviewees

Job titles of interviewees varied, but the most common were Director of Nursing, Director of Patient and Public Involvement, Quality Development Manager and Head of Clinical Governance. Most interviewees had been involved with the surveys since 2001.

### Attitudes towards patient surveys

About half of the respondents noted that the national patient surveys provided reliable, credible and fair benchmarks against other NHS trusts, and that survey results strengthened the validity and usefulness of other information on patient experience: “The results do reinforce where we suspect there are problems.”

The fact that the surveys were repeated regularly was welcomed because it facilitated longitudinal comparisons and meant they were well-established as performance measures: “People have taken notice since it’s clear that it’s a rolling programme and we will be able to measure whether we’ve made changes.”

Almost all interviewees said that the surveys were more widely accepted now than when they first started, and some said that their own attitudes had become more positive: “It would be awful if the surveys were stopped now. It takes 2–5 years for a new initiative to be accepted. The surveys are really starting to get accepted now.”

There were concerns that the surveys were not good value for money. Six interviewees said that they did not provide new information, but just confirmed what was already known: “There are rarely any surprises with the patient survey.”

### Uses of patient survey results

Almost all interviewees said they used patient survey results as a basis for action plans aimed at improving the quality of care and for measuring the success of those plans. About a quarter of them also said that the implementation of action plans was now part of some individuals’ performance assessment.

However, four interviewees said that they had learned that they needed to take a more directive approach to implementing action plans: “Just giving people the results doesn’t mean they will take action. They need direction to make them do things and the frameworks to help them.”

The generally positive surveys findings were mentioned by five interviewees as being helpful in boosting staff morale.

### Examples of quality-improvement interventions

Although particular quality improvements made by trusts were not explored in depth in this research, some mentioned innovative ways in which they had used patient experience information. For example, one interviewee said that the patient survey highlighted a problem with noise at night, so they had asked patients to keep records of the source of disturbing noises. As a result, floor coverings were changed, quieter waste bins were installed, and, where possible, patients admitted overnight were put into a separate area. Another trust’s patient survey results had prompted them to recognise that their provision of discharge information was patchy, so they produced comprehensive discharge information packs, which were given to patients on admission.

### Comparison of sources of patient experience information

Fifteen respondents said they found the patients’ comments written on questionnaires particularly interesting and useful for getting clinicians’ attention: “Reading through the comments, even though our percentage scores are OK, you think, ‘That shouldn’t have happened.’”

There was also support for the more qualitative feedback methods, although it was recognised that these were more labour-intensive and could be impractical for gathering systematic feedback. The most popular sources were complaints, prioritised by nine respondents, “Complaints really tell you how it is,” and PALS by six, followed by the national patient surveys, which were given the greatest weight by five interviewees.

Comments cards and suggestion boxes were thought useful because they offered immediate feedback. The robust nature of the patient surveys was cited as an important factor: “Without a doubt, the national patient surveys are given the most weight. We have nothing else that is so sophisticated and would give us such useful data.”

Six respondents commented on the importance of looking at different sources of patient experience information concurrently. The surveys were seen as adding harder evidence to “soft” information, such as comments or complaints, while they also added patient experience information to “hard” clinical or routine data.

### Quality of questionnaires and methods

Fourteen respondents said the quality of the questionnaires was good, and three also mentioned that they used the national surveys as models for constructing their own local questionnaires, rather than designing their own surveys: “Everyone thinks they can design a questionnaire, so it’s cut down on badly prepared surveys.”

The most common concern about questionnaires, mentioned by five respondents, was that they were too long.

Almost all respondents were confident in the rigour of the survey methods. Some were concerned that the sample size was too small, but most of those concerns seemed to be based on a misunderstanding that sample size should necessarily be proportionate to population size: “I often find that clinicians will say that it isn’t representative, as the numbers are very small in comparison to the numbers of patients we treat.”

### Dissemination of survey findings

Results were communicated to patients and the public through posters and leaflets in public areas, press releases, annual reports, presentations to patient and public involvement groups and articles in the trust’s magazines. The most common way of disseminating survey results to staff were the organisation’s intranet, newsletters, meetings at which approved contractors presented results, teaching sessions and special events designed to engage staff in forward planning.

In most organisations, results were sent to senior staff, who were expected to cascade results down to junior staff. It was recognised that some groups of staff, such as doctors or more junior staff, were less likely to receive survey results: “If I could change anything … I would be better at sharing results and information about what we’re doing in response to them.”

Some interviewees said their boards did not receive patient survey results, some sent a summary report to the board, and in others the trust board received detailed information about the survey results in the form of both written materials and presentations.

### Incentives, barriers and solutions

Interviewees noted a number of factors which affected their use of the patient surveys in positive or negative ways, and several had tried to implement local solutions or suggested improvements to the national programme.

#### Specificity of results

The most commonly cited barrier to using survey results was that the feedback was not specific enough to be salient to those who needed to act on it. That is, clinicians, particularly doctors, were interested mainly in their own sphere of influence: “The main criticism we have from doctors is ‘Make it specific to the area I work in and I will take notice of it.’”

For the 2006 inpatient survey, trusts were required to include information on patient’s specialties in the data they submitted to the Healthcare Commission, so we asked whether they planned to analyse their results by specialty. Some interviewees had already done this, or planned to do it, while others liked the idea but had not yet thought of it.

#### Clinicians’ engagement with the surveys

A few respondents mentioned that clinical staff questioned the validity of the surveys. One comment underlined this view: “Sometimes doctors and nurses question their validity but some people would rather look at anything but the issues raised by the questions.”

Several respondents said that there was variation among clinicians in their receptiveness to survey results. Some found that nurses were easier to engage than doctors. Several had tried ways of improving clinician engagement, including seeking opportunities to present the results to clinicians. Training and induction programmes were used as opportunities to share survey results.

#### Culture

The importance of taking account of patients’ views was a common theme, and there was a sense that this was still a fairly new approach to planning services, and that a culture change was under way. One of the most commonly cited reasons for using patient survey results was that the culture of the organisation, and their Chief Executive, supported it.

#### Difficulty in knowing what to do next

Some interviewees said they found it difficult to identify the reasons behind their successes or failures, or in knowing what to do to make improvements. The importance of spreading best practice in service improvement is noted in *Delivering the NHS Plan.*^19^ When asked, about half of the interviewees said they would be interested in knowing how others had made improvements, or in identifying high scorers so that they could learn from them: “We work in a *National* Health Service so that’s exactly what we should be doing: sharing best practice.”

However, despite complaints that they did not know what to do to make improvements, several interviewees were not particularly enthusiastic about proposed strategies for identifying best practice. On the other hand, a surprising number of others, who said they had not thought of looking at other organisations’ performance, or did not know how to do it, said it would be a good idea.

#### Time and resources

Lack of time and resources, and competing demands on their time were mentioned by seven interviewees as inhibiting factors in implementing quality improvements.

#### Targets, financial incentives and external assessments

A number of respondents thought external incentives, particularly the annual published performance ratings, could be important stimuli to increasing the focus on patient surveys: “If [the surveys] were heavily weighted in the annual performance ratings, they would get more attention.”

Six said financial incentives, including PbR, were important drivers for change. Targets evoked mixed feelings, often within the same individuals, who tended to say they did not like targets, and were concerned that they diverted resources away from important services, but conceded that they were strong drivers for change.

The Patient Choice Agenda^20^ was named by several as an important factor in driving quality improvement: “Clinicians aren’t really sceptical any more. What patients think of us is increasingly important because of choice.”

### Reporting of survey results

Opinions on the Healthcare Commission’s presentation of published results were almost universally positive, particularly regarding the benchmark “traffic light” charts, which show in three-colour bands whether their trust’s score on each question falls within the best 20% of trusts, the worst 20% or the middle 60%: “With the simple traffic lights, you can see quite clearly where you are and where you should be.”

Most respondents were interested both in comparing their own trust’s performance with previous years, and in comparing their current performance with other trusts, but there was slightly more interest in their own organisation’s change over time. Many commented that the surveys have become more useful now that year-on-year comparisons are possible: “We are interested to see if our actions have been effective, and if any areas have gone up or down.”

One person commented that the prompt reporting of the patient survey results was unlike most other centrally managed audits, but another said the time between the survey being carried out and the results being published was too long: “If the benchmarks came out earlier, it would help us to act on them more immediately.”

Related to this issue were concerns that the surveys covered only a short and very specific time frame, and that fluctuations in the quality of care might not therefore be adequately reflected in the results: “It’s a pretty blunt instrument; it only captures a moment in time: a small number of people’s experiences.”

**Box 1 Barriers to using survey results**

- Data were not specific enough to wards, departments or specialties
- Lack of time and resources
- Not knowing what to do about the survey results
- Lack of statistical expertise

**Box 2 Facilitators for using survey results**

- Survey results made an important contribution to the organisation’s performance ratings
- A patient-centred organisational culture
- Detailed and clear benchmark information
- Repetition of the same surveys, facilitating longitudinal comparisons

**Box 3 Recommendations for improving patient survey programmes**

- Repeat the same surveys at regular intervals
- Run regular workshops to facilitate networking and educate survey leads
- Disseminate information about the basic statistics relevant to patient surveys
- Gather data on smaller units and/or encourage organisations to analyse their existing results by smaller units
- Give patient surveys prominence in performance-management systems
- Continue to publish benchmark charts in a “traffic light” format
- Ensure that results are published quickly after completion of surveys
- Ensure that a section for patient comments is included in questionnaires
- Consider collecting patient survey data at more regular intervals

## DISCUSSION

Patient survey results were judged to be accurate and robust indicators of patient experiences. Almost all respondents said attitudes had become more positive since the survey programme began. Interviewees were complimentary about the standard of the questionnaires and the survey methods, and particularly liked the patients’ written comments. Consistent with previous UK research,^9^ complaints and PALS information were valued more highly than the surveys in some respects, but the surveys were generally thought to be more robust. Several interviewees emphasised the importance of integrating survey results with other patient experience information such as complaints and comments cards to validate, triangulate and illustrate findings.

Consistent with previous research, the most common barrier to using survey findings was that results were not specific enough to smaller units within the organisation. There were also suggestions that more continuous feedback, rather than an annual “snapshot,” would be useful. A lack of knowledge of effective interventions and lack of time were also important barriers. There was some scepticism, particularly among clinicians, about the validity of the survey results. Sample sizes were a concern, but there were some statistical misunderstandings. The low priority given to using survey results within the NHS was thought to be a barrier to their use.

Many of the findings on incentives for using the results also concurred with previous research. Several respondents said that targets, the surveys’ weightings in performance ratings and financial incentives were, or would be, important in strengthening their impact, but they also said that they did not like targets. On the other hand, many mentioned internal drivers, such as a desire to deliver high-quality patient-centred care, as important in stimulating work with patient surveys. The patient surveys, along with leadership by senior managers, were thought to be important promoters of a patient-centred culture. Almost all respondents were positive about the reporting by the Healthcare Commission of inter-organisational benchmarks for each NHS trust on each survey question. This detailed information was of particular interest for making longitudinal within-trust comparisons.

We did not replicate a previous finding that the time lag between survey administration and reporting is a barrier to their use.^9^ ^10^ It is possible that this is because, for most of the national surveys in England, results have been published within 6 months of their administration, and many trusts receive results earlier than that from their approved contractors. Also, there was only moderate enthusiasm for the provision of information on high-performing trusts and on successful quality improvements, suggesting that a “systematic approach to quality improvement” is not necessarily recognised as valuable.

It was interesting that most of those who had not thought of analysing their own survey results by smaller units were positive about the idea. Similarly, identifying high scorers so that lessons could be learned from them was a new idea for many, some of whom thought it was a good idea. This suggests that disseminating knowledge about the survey methods and better networking arrangements for survey leads would be beneficial.

The non-probablility sampling method used in this study, and its relatively small scale, meant that it was not possible to ensure that every subgroup of trust type was included in the sample. However, the sampled trusts were broadly representative of the population in their size, performance ratings and location within or outside London. Furthermore, it would have been impractical to employ a probability sampling method, given the large number of potential confounding organisational variables, the lack of information about the influence of organisational variables on attitudes towards patient surveys and the qualitative nature of the study.

It is possible that employees in trusts with more positive attitudes towards the surveys were more willing to participate, and this could have introduced a self-selection bias. This is perhaps why all of the selected three-star trusts participated. However, to minimise bias, once a trust had been selected, considerable efforts were made to obtain an interview, and the resulting response rate was high.

## CONCLUSION

Many of the attitudes and beliefs about the current national patient survey programme in England are positive, one of its key strengths being the surveys’ regular repetition. This research highlighted a number of issues that are important for the success of patient survey programmes, including the need to make results specific to smaller units, strengthening the survey results’ profile in performance assessments and facilitating networking for those involved with the surveys.
